# Patients' Experiences of Web-Based Access to Electronic Health Records in Finland: Cross-sectional Survey

**DOI:** 10.2196/37438

**Published:** 2022-06-06

**Authors:** Sari Kujala, Iiris Hörhammer, Akseli Väyrynen, Mari Holmroos, Mirva Nättiaho-Rönnholm, Maria Hägglund, Monika Alise Johansen

**Affiliations:** 1 Department of Computer Science Aalto University Espoo Finland; 2 Department of Industrial Engineering and Management Aalto University Espoo Finland; 3 Kela, The Social Insurance Institution of Finland Helsinki Finland; 4 Healthcare Sciences and e-Health, Department of Women's and Children's Health Uppsala University Uppsala Sweden; 5 Norwegian Centre for E-health Research University Hospital of North Norway Tromsø Norway

**Keywords:** patient portals, EHR, electronic health record, open notes, patient access, self-management, national survey

## Abstract

**Background:**

Patient portals that provide access to electronic health records offer a means for patients to better understand and self-manage their health. Yet, patient access to electronic health records raises many concerns among physicians, and little is known about the use practices and experiences of patients who access their electronic health records via a mature patient portal that has been available for citizens for over five years.

**Objective:**

We aimed to identify patients’ experiences using a national patient portal to access their electronic health records. In particular, we focused on understanding usability-related perceptions and the benefits and challenges of reading clinical notes written by health care professionals.

**Methods:**

Data were collected from 3135 patient users of the Finnish My Kanta patient portal through a web-based survey in June 2021 (response rate: 0.7%). Patients received an invitation to complete the questionnaire when they logged out of the patient portal. Respondents were asked to rate the usability of the patient portal, and the ratings were used to calculate approximations of the System Usability Scale score. Patients were also asked about the usefulness of features, and whether they had discussed the notes with health professionals. Open-ended questions were used to ask patients about their experiences of the benefits and challenges related to reading health professionals’ notes.

**Results:**

Overall, patient evaluations of My Kanta were positive, and its usability was rated as good (System Usability Scale score approximation: mean 72.7, SD 15.9). Patients found the portal to be the most useful for managing prescriptions and viewing the results of examinations and medical notes. Viewing notes was the most frequent reason (978/3135, 31.2%) for visiting the portal. Benefits of reading the notes mentioned by patients included remembering and understanding what was said by health professionals and the instructions given during an appointment, the convenience of receiving information about health and care, the capability to check the accuracy of notes, and using the information to support self-management. However, there were challenges related to difficulty in understanding medical terminology, incorrect or inadequate notes, missing notes, and usability.

**Conclusions:**

Patients actively used medical notes to receive information to follow professionals' instructions to take care of their health, and patient access to electronic health records can support self-management. However, for the benefits to be realized, improvements in the quality and availability of medical professionals’ notes are necessary. Providing a standard information structure could help patients find the information they need. Furthermore, linking notes to vocabularies and other information sources could also improve the understandability of medical terminology; patient agency could be supported by allowing them to add comments to their notes, and patient trust of the system could be improved by allowing them to control the visibility of the professionals’ notes.

## Introduction

Patient portals that provide access to electronic health records (EHRs) are becoming increasingly common. Such access to EHRs offers the means for patients to better understand personal health issues, treatment plans, and decisions [1], thus supporting personal health management [2] and informing patients between time- and resource-consuming clinic visits or phone appointments [3].

“Open notes,” which are clinical notes that are shared with patients [4], can be considered an essential part of any patient-accessible EHR. In some countries, for example, Sweden [5], Norway [6], and Finland [7], nationwide patient-accessible EHR services, including open notes, are offered to most citizens through national patient portals.

Moreover, the majority of studies in recent reviews [3,8,9] highlighted benefits of patient access to EHRs. Patients were satisfied with the communication and engagement with clinicians, as well as better self-care, achieved as a result of patient access [8]. Improved doctor–patient relationships and patient outcomes were also found to be benefits [3].

Despite these benefits, health care professionals often criticize patient access to EHR [10]; patients, on the other hand, would like more doctors to offer access to their notes [11]. Patient access to EHR changes the physician–patient relationship and power dynamic; physicians have raised concerns [10,12] that such access may worry patients, cause misunderstandings, or cause extra work for physicians [13,14]. Physicians have also been worried that patients who find mistakes or errors would call and ask for corrections to notes which would increase the workload for health care [10].

Many studies [15-18] have also reported lower than anticipated levels of patient uptake of EHR access. Thus, in order to realize the potential of such access to support patient self-management, a better understanding of patient practices, motivations, and challenges is necessary. As de Lusignan et al [15] pointed out, there is still a need to understand how web-based access to EHR might be “redesigned to guide and teach patients in a way that promotes self-management and ultimately improves health.”

Patient experiences with access to EHRs have often been explored using surveys, whereby patients were asked to rate usability [19] and attitude [5], usefulness [6], ease of use [20,21], and benefits and risks [22]. In addition, Bell et al [23] used a Likert-scale to study how reading notes affected patient–doctor relationships. Qualitative data have also been collected to understand patient views of access to EHRs. Mishra et al [24] included open-ended questions to identify positive and negative themes related to the usefulness, understandability, and worries caused by patient access; Gerard et al [25] used open-ended questions about the value of reading notes and providing feedback on open notes; Rexhepi et al [26] interviewed patients with cancer and found that patient access helped them prepare for doctor visits and understand their medical issues; and Eriksson-Backa et al [27] conducted focus groups with older adults and identified the uses, enablers, barriers, and behavioral outcomes of the national My Kanta patient portal.

In Finland, My Kanta, a nationwide patient portal, was introduced in 2010 and varied functions were adopted in a step-by-step manner [28]. Since 2015, the My Kanta patient portal has enabled all citizens using public health care services to access their health records and prescriptions, and to renew the latter [28]. The use of My Kanta is very established, with 63% of Finnish adults having accessed the patient portal during the period from 2010 to 2018 [7], and 92% of adults (from 18 to 65 years) used the patient portal in 2021. The most used functions among pharmacy customers were browsing prescription information (97.4%) and health records (96.3%) [20].

The goal of this study was to understand patients’ experiences using My Kanta to access their EHRs. While My Kanta has been available for all patients to use for 7 years, little is known about patient use practices and experiences. Thus, we specifically focused on understanding patients’ perceptions related to the usability of the patient portal and the benefits and challenges of reading the clinical notes written by health care professionals.

## Methods

### Design

We conducted a cross-sectional survey to capture patients’ experiences using the My Kanta patient portal.

### The My Kanta Patient Portal

My Kanta is a web-based patient portal for all residents with a Finnish personal identity number and access to electronic identification. Patients can view their own or their dependents’ health data (consisting of records of health care visits, diagnoses, critical risk factors, laboratory tests, x-ray examinations, referrals, health and care plans, and medical certificates, statements [20], and e-prescriptions), request a prescription renewal, and save living wills and organ donation testaments [29].

My Kanta is a part of national Kanta services that integrate and save medical, health, and prescription data for health care providers, citizens, and pharmacies [28]. All public and private health care providers that use electronic patient record systems are obliged by law to send prescription and health data to Kanta services [7]. Health data, test results, and prescriptions can be used by health care units with patient consent [28], which can be given or withdrawn on My Kanta.

According to international benchmarking, My Kanta provided patients and their caregivers with the best access to their health record data alongside Korea in 2019 [30] and also provided the most functions in 2016 [31]. However, My Kanta does not allow typical patient portal functions, such as appointment booking or communication with health care professionals.

### Questionnaire

The web-based questionnaire included 4 open-ended questions and 11 questions with Likert scale or multiple choice response options (Multimedia Appendix 1). The topics of the questions were (1) reasons for logging into the patient portal and whether the visit was successful or not and why; (2) subjective usability of the patient portal; (3) usefulness of the features of the patient portal; (4) the benefits and challenges of reading health care professionals’ notes and discussing their notes with them; (5) improvement ideas for the patient portal; (6) guidance on reading the notes; and (7) background information.

To assess perceived usability, a 2-item questionnaire based on the Usability Metric for User Experience [32]—the UMUX-LITE scale [33]—was used. UMUX-LITE scores were transformed, using a corrective regression formula [33], to System Usability Scale scores. The System Usability Scale is the most frequently used questionnaire for measuring the subjective usability of eHealth apps [34]. Borsci et al [35] tested UMUX-LITE with health care professionals and found it to be appropriate for use in the context of health care technology [35].

Open-ended questions about respondents’ experiences of the benefits and challenges of reading health care professionals’ notes were used in order to collect qualitative data about the most relevant issues from the patients’ perspectives. The web-based questionnaire was dynamic; only respondents who reported having read the notes at least once (ie, had actual use experience) were asked the open-ended follow-up questions. If a respondent rated reading the notes as “not useful,” they were only asked about challenges (to avoid unnecessarily asking these respondents questions about benefits). The survey was available in both official languages of Finland: Finnish and Swedish.

The questionnaire was reviewed by 2 researchers in the field and 2 experts from the Social Insurance Institution of Finland, which was the organization responsible for developing My Kanta. In addition, we pilot-tested the questionnaire with 3 patients who filled in the questionnaire and simultaneously talked aloud about how they understood the questions. The questionnaire was subsequently revised to clarify wording.

### Conducting the Survey

Data were gathered during the period from June 4, 2021 to June 14, 2021 using a web-based questionnaire. Patient users of My Kanta in Finland received an invitation and a link to the questionnaire when they logged out of the patient portal. Thus, all respondents had used the patient portal just before they responded to the questionnaire. Participation was voluntary and anonymous.

### Ethics Approval

The study protocol was reviewed and approved by the Ethical Review Board of Aalto University (ethics approval number D/957/03.04/2020 Nordic eHealth for Patients).

### Analysis

Descriptive statistics were calculated for quantitative data (respondents’ characteristics: age, gender, and portal usage). We performed content analysis (Atlas.ti, version 8.4.5; ATLAS.ti Scientific Software Development GmbH) on the responses to open-ended questions. One researcher first read through the data and used open coding to identify themes in the data without predefined categories. Short sentences were chosen as the analytical unit; themes were defined using in vivo coding, and to ensure that the themes represented the original meaning of the respondents, we used respondents’ sentences to label the themes. The number of respondents who mentioned a theme was calculated, and the themes were categorized. A second researcher then reviewed the results. The researchers discussed similarities and differences in themes and combined categories, until a version was agreed upon as the final version.

## Results

### Respondents

Of 449,922 users who logged in, 3139 users responded to the survey (response rate 0.7%). Most users reported either weekly (889/3112, 28.6%) or monthly use (1120/3112, 36.0%) (Table 1). The frequency of use was comparable to that of My Kanta in May 2019, when users used My Kanta on an average of 2.4 times per month [7]. The proportion of users over the age of 50 years was high (2681/3135, 85.5%). The proportion was 2-fold that in 2021 (44%). Although the frequency of use may vary notably between users, this may suggest overrepresentation of older age groups among respondents.

**Table 1:** Respondent characteristics (n=3135).

Characteristic			Respondents, n (%)
**Gender (n=3118)**			
	Female	2104 (67.5)	
	Male	962 (30.8)	
	Other	52 (1.7)	
**Age (years) (n=3115)**			
	<18	5 (0.2)	
	18-35	93 (3.0)	
	36-50	336 (10.8)	
	51-65	1082 (34.7)	
	66-75	1173 (37.6)	
	76-85	395 (12.7)	
	>85	31 (1.0)	
**Frequency of use (n=3112)**			
	Daily	194 (6.2)	
	Weekly	889 (28.6)	
	Monthly	1120 (36.0)	
	Less than once per month	878 (28.2)	
	First time user	31 (1.0)	
**Success of the visit (n=3125)**			
	Yes	2247 (71.9)	
	No	766 (24.5)	
	Do not know	112 (3.6)	
**Device used (n=3053)**			
	Computer	1836 (60.1)	
	Smartphone	690 (22.6)	
	Tablet	522 (17.1)	
	Something else	5 (0.2)	
**Has discussed the notes with a health care professional (n=3039)**			
	Yes	1046 (34.4)	
	No	1993 (65.6)	

### Experiences With the Patient Portal

The total mean score for the System Usability Scale approximation was 72.7 (SD 15.9).

The most common reasons for visiting the My Kanta patient portal were viewing medical notes (978/3135, 31.2%), results of examinations (693/3135, 22.1%) or prescriptions (548/3135, 17.5%). Many people also visited the patient portal to renew a prescription (477/3135,15.2%), because there is no other method for renewing prescriptions electronically. At the time of the survey, COVID-19 vaccinations had started in Finland, and many (229/3135, 7.3%) logged into the patient portal to view their vaccination certificates. Other functions were used by only a few respondents (n=6-21). Some users (n=24) tried to use functions that did not exist, such as making appointments or checking their appointments (n=18), contact health care professionals (n=4), or looking for information about the reason that their prescription had not been renewed (n=2).

**Figure 1:**
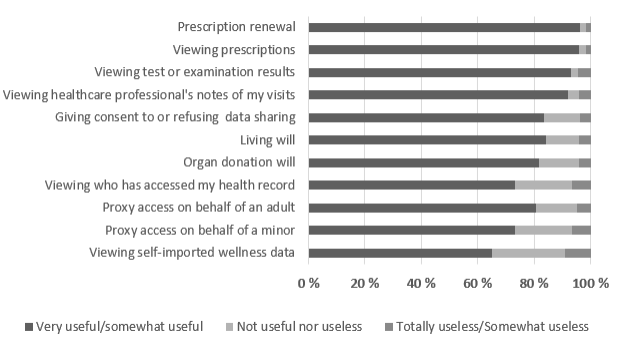
Usefulness of My Kanta patient portal features.

The most used functions were also deemed to be the most useful (Figure 1); for example, 96.4% (2511/2605) of users considered prescription renewal and 91.9% (2749/2992) of users considered viewing health care professionals’ notes to be very useful or somewhat useful; however, the majority of users also considered less rarely used functions useful, with the lowest percentage (759/1165, 65.2%) of users considering self-reported wellness data, and the highest percentage (1483/1759, 84.3%) of users considering living will to be very useful or somewhat useful.

### Benefits and Challenges of Reading Notes

Most respondents (2183/3135, 69.6%) answered the open-ended question and mentioned one or more benefits of reading notes (Table 2). Of the perceived benefits, most often respondents stated (560/2178, 25.7%) that notes supported remembering things:

One can recall afterwards what happened in the health care visit and what was discussed about.

Respondents often mentioned that they felt tense or overwhelmed during their appointment, and notes helped in remembering what was said and which instructions were received.

**Table 2:** Themes of perceived benefits of viewing medical notes.

Benefits			Mentions (n=2178), n (%)
**Supports remembering**			560 (25.7)
	What a doctor or a nurse said	505 (23.2)	
	The care history	55 (2.5)	
**Provides information**			495 (22.7)
	About health and care	223 (15.0)	
	To check the state of health and remain up-to-date	74 (3.4)	
	On how I and my disease are perceived	70 (3.2)	
	On all information concerning myself	43 (2.0)	
	That is more detailed and was not said during the appointment	34 (1.6)	
	About what was done during an appointment	30 (1.4)	
	On diagnoses	21 (1.0)	
**Convenience of patient portal access**			449 (20.6)
	Ability to return to view all the saved information	155 (7.1)	
	Can be checked at leisure	98 (4.5)	
	No need to call or contact health care	73 (3.4)	
	Easy of finding information	57 (2.6)	
	Fast access	56 (2.6)	
	Clear and reliable information	10 (0.5)	
**Helps in understanding**			339 (15.6)
	Own condition or what was said	326 (15.0)	
	Whether more can be asked if something was unclear	13 (0.6)	
**Ability to check the notes**			234 (10.7)
	Identifying potential errors and misunderstandings	142 (6.5)	
	Asking for error corrections	65 (3.0)	
	Checking that all essential information was written	21 (1.0)	
	Increases transparency and reliability	6 (0.3)	
**Supports self-management**			175 (8.0)
	Checking the care plan and next steps	63 (2.9)	
	Following the course of care success	36 (1.7)	
	Preparing for the next appointment	29 (1.3)	
	Looking for further information	14 (0.6)	
	Helps in communicating with health care professionals, learning to express yourself	14 (0.6)	
	Supports self-care	13 (0.6)	
	Enables peace of mind	6 (0.3)	

Respondents appreciated that notes provided information about their health and care. They were able to follow the course of their care and remain up-to-date. Furthermore, they wanted to identify doctors’ perceptions of them and their diseases. Several mentioned that it is important to have all the information concerning themselves:

My life and my own information are certainly of primary importance.

Respondents also noted that the information is provided conveniently in one place, and they can check the information whenever they want. Notes were also perceived as helping them to understand their health conditions and what health care professionals had said during appointments. In addition, many respondents wanted to check the notes to ensure there were no errors or misunderstandings.

Many stated that the reason for accessing the information and remaining up-to-date was to actively self-manage their health. Respondents wanted to be aware of their care plans and to follow the course of their success. They subsequently prepared themselves for the next appointment and looked for further information related to their condition and care. A few commented that the notes helped in communicating with health care professionals and supported learning to express themselves, and 1046 out of 3135 (33.4%) respondents also discussed the notes with health care professionals.

One-third (1175/3135, 37.5%) of respondents also reported one or more challenges in reading notes (Table 3). The most commonly mentioned challenge was the difficulty in understanding the notes and the medical terminology. For example, one respondent stated:

Language that I don’t understand. Wikipedia may help in translation work, when you don’t understand the crucial words.

Many mentioned that they used Google to interpret the unfamiliar terms, codes, and abbreviations, and they wanted plain language to be used instead.

**Table 3:** Perceived challenges of viewing medical notes.

Challenges		Mentions (n=1175), n (%)
**Notes are difficult to understand**		707 (60.2)
	The medical terminology is difficult to understand	523 (44.5)
	Abbreviations are difficult	73 (6.2)
	Examination and test results are difficult	44 (3.7)
	Notes in general are difficult to understand	44 (3.7)
	Diagnoses are not understandable	23 (2.0)
**Notes are not available**		232 (19.7)
	Delay in access	121 (10.3)
	Missing information	105 (8.9)
	Children’s information is not visible	6 (0.5)
**Notes are incorrect or inadequate**		217 (18.5)
	Incorrect information or errors	80 (6.8)
	Health care professionals’ misinterpretations	28 (2.4)
	Imprecise notes	27 (2.3)
	Very brief notes	16 (1.4)
	Negligent writing	15 (1.3)
	Irrelevant or too detailed information	12 (1.0)
	Repetition	10 (0.9)
	Poor language	9 (0.8)
	Wrong language (eg, Finnish instead of Swedish)	7 (0.6)
	Too personal	5 (0.4)
	Inappropriate	4 (0.3)
	Follow-up is unclear	4 (0.3)
**Problems with usability**		167 (17.4)
	Information was difficult to find	85 (7.2)
	Errors are difficult or impossible to correct	37 (3.1)
	Could be easier to use	25 (2.1)
	Disorganized	25 (2.1)
	No interactivity	8 (0.7)
	The search process is cumbersome	5 (0.4)
	Worries about privacy	5 (0.4)
	Comparing examination results is difficult	5 (0.4)
	Reading on mobile devices is difficult	5 (0.4)
	The text is small	4 (0.3)

However, the notes were not always available because there were delays in access and some visits were not recorded or visible. It was mentioned that it could take days or weeks before the notes were available, and some information was not available at all.

Many respondents perceived notes to be incorrect or inadequate. Most commonly, they were seen as having errors—some were not significant, such as a wrong date, but some were more severe, such as having a wrong diagnosis or another patient’s information. Respondents described,

Mainly the challenge is that the communication has been wrongly recorded or it is misunderstood. People should have possibility to say their views on My Kanta

and

Sometimes there have been erroneous information and diagnoses. For example, a cancer that I don’t have.

Many also reported that the notes differed from what they had experienced themselves. Several also wished for more detailed notes. In contrast, some felt that it was unnecessary to include all personal details that they had mentioned during an appointment or the whole message that they had sent. One person also mentioned that they did not want to talk about certain issues, because they would be recorded and seen by all professionals.

Finally, there were challenges related to the usability of the system. Most commonly, it was mentioned that it was difficult to find information. The information was not always in chronological order, and some examination results were not linked to the appropriate appointments. A few respondents also mentioned that there is no interactivity in the system, and they wanted to comment on the notes or request corrections. Furthermore, it was noted that a patient should receive a notification when new information is available.

## Discussion

### Principal Results

Respondents evaluated the My Kanta patient portal as useful and usable, which is consistent with the findings of earlier studies [20,21]. The total mean score for the System Usability Scale approximation was 72.7 (SD 15.9), which can verbally be described as good usability, according to Bangor et al [36,37]. Prescription renewal and viewing were indicated to be the most useful functions, but viewing medical notes and the results of examinations were the most frequent reasons for visiting the patient portals, which 91.9% (2749/3135) and 92.9 % (2770/3135) of respondents, respectively, considered useful.

Furthermore, respondents explained in their responses to open-ended questions that they appreciated having access to EHRs and information via a patient portal, which supports earlier findings [6,22]. Because My Kanta has been used nationally for several years, respondents were already familiar with the portal and actively used medical notes to prepare for their communications with health care professionals and to take care of their health.

The qualitative responses provided a rich and versatile description of the benefits of patient access to EHRs. Specifically, reading the notes was described as convenient, because they could be accessed easily and quickly, whenever suitable and at leisure. Therefore, easy access via patient portals may help patients to be engaged in self-management of their health. Reading notes were described as supporting remembering and understanding what health professionals said. They were able to check the state of health and care plans, remain up-to-date, look for further information, prepare for the next appointment, and ask further questions if something was unclear. We suggest that these activities support patients in learning about their disease or care, which motivates them to take care of their health.

Furthermore, reading notes can provide information that is not directly addressed during visits with a health care professional. As previously suggested [38], this may improve patient autonomy by reducing dependence on individual health care professionals and providing the opportunity to consult medical literature or other health care professionals to better understand health status and options for care or treatments.

Many respondents stated that it was important to be able to check the notes to identify potential errors and misunderstandings. They were also interested in professionals’ perceptions of their situations. Reading the notes was thus seen to help them understand what health care professionals had said and prepare for the next appointment. Thus, patient access to EHR supports patient–provider communication.

Very few patients were concerned about privacy or felt the notes were too personal or inappropriate. Some patients found incorrect information, and a few mentioned serious errors. It was very rarely mentioned, but a few respondents also felt that the notes included irrelevant information or personal information that was too detailed. Although rarely mentioned, the notes sometimes included information about very personal issues that patients were unwilling to share with all health care personnel. In particular, when a patient portal does not allow patients to correct errors or express their views with a comment, we presume that some patients may feel that their self-determination is violated.

It is noteworthy that respondents did not perceive reading the notes to be harmful per se but that challenges, such as understandability of medical terminology, incorrect or inadequate notes, missing information, or difficulties in finding information, interfered with the benefits of reading the notes. Finnish law requires that professionals’ notes are sufficiently comprehensive, clear, and understandable and that only commonly known terms or abbreviations are used [39]. Nevertheless, this is clearly not fulfilled according to the survey results.

In order to realize associated benefits, improvements in the quality and availability of medical professionals’ notes are needed. In addition to educating health care professionals, the availability of information can also be supported by providing a standard information structure. Because the information structure was confusing to patients, a standard structure would make finding and reading information easier from patients’ perspectives. It is important that the order of the notes is logical from their point of view and that examination results are clearly linked to corresponding appointments. Linking the notes to vocabularies and other information sources could also improve the understandability of medical terminology without increasing the workload of professionals. In addition, patient agency and trust could be supported by enabling them to add comments to their notes, mark some entries as sensitive, and control the visibility of entries.

### Limitations

This was a cross-sectional survey study examining patients’ self-reported experiences of the national patient portal in Finland, and the results may not be generalizable to other countries or patient portals. The survey was available only to My Kanta users after logging out of the patient portal. Not all users may have actively logged out the portal or noticed the invitation, which may have contributed to the low response rate. Thus, the results do not represent all My Kanta users or the population of Finland. A similar survey study in Sweden [5] also had a low response rate (0.61%).

In addition, the only demographic information available from the survey was age and gender; health and socioeconomic status of the respondents, literacy, and health literacy remained unknown. It is possible that the survey respondents represented users who were most interested in the patient portal and most capable of using it. Our sample did not include persons who had stopped using the portal or were not able to use it. Thus, nonrespondents may differ in their use of the patient portal and may experience barriers (eg, [40,41]) that were not identified in this study.

Moreover, My Kanta includes many functions that were recently added and, thus, not widely used. Therefore, the usefulness of all the functions could not be reliably evaluated. Furthermore, the portal does not have all the potentially useful functions that users could have experienced. As a few respondents complained, My Kanta does not have much interactivity—patients are not allowed to comment on notes or request corrections in the portal. In addition, the lack of notifications on added content frustrated respondents, because they often logged in to look for notes or test results that were not available yet.

By asking open-ended questions on the benefits and challenges, we improved the reliability of the answers as respondents reported their experiences using their own words and were not guided by having to choose from certain options. Because the number of respondents was high, the data that we collected were rich and versatile. However, respondents may have focused on the most significant benefits and challenges they experienced, and they may not have been able to verbalize those that were more abstract and less obvious. Therefore, we believe that our mixed methods survey study complements previous quantitative studies [5,6,19-23].

### Comparison With Prior Work

The main benefits experienced by patients were very similar to those identified in a smaller study [25] conducted at a single institution in the United States over a 12-month pilot period, in which participants reported that reading notes helped them to better remember next steps, provided positive emotions, and gave them faster access and results. The participants in the study [25] also valued the opportunity to correct any possible misunderstandings and give feedback to their providers, which are functions that patients also wished were available on My Kanta. In addition, Rexhepi et al [26] and Pyper et al [42] identified similar benefits, in studies in Sweden and the United Kingdom, respectively. Thus, our study provides further details in understanding self-management practices that patient access to EHR can support.

Moreover, several studies [6,20,24,26,27,42-44] have identified that some parts of medical records are difficult to understand. In addition, Johansen et al [14] found that 25.6% of administrative staff and 15.4% of health care professionals had received feedback from patients or their relatives regarding mistakes or missing information in their EHR. In our study, the number of serious mistakes was seldom mentioned, which was not the case in a recent US survey [45] with 29,656 respondents, in which 1 in 5 patients reported finding a mistake, and 40% perceived the mistake to be serious. It is possible that the number of respondents who found errors would have been higher in our study if we had specifically asked about this. This should be explored in future studies.

Pyper et al [42] also found that patients identified errors and omissions and had differences of opinion when they accessed electronic records for the first time. In this context, our study shows that unclear or inadequate notes are very common even when patients are familiar with use of the EHR and the challenges do not disappear when health care professionals gain experience in conveying information to patients. Thus, the findings support the need for applications that provide explanations of medical terms in EHR notes (eg, [46,47]).

Thus, patients’ basic needs and self-management processes seem to be similar regardless of the context—benefits and challenges experienced by patients are remarkably similar across countries, different health care systems, and EHRs.

### Conclusions

Our findings indicate that patient access to EHR can support self-management—patients actively used medical notes to understand and remember what health care professionals said and to take care of their health. The challenges interfered with the benefits of reading the notes. In order to realize benefits, improvements in the quality and availability of medical professionals’ notes are needed, and patients should be encouraged to discuss their concerns with them. In addition, the availability of information can also be supported by using a standard information structure. Specifically, linking the notes to vocabularies and other information sources could also improve the understandability of medical terminology.

## Appendix

Multimedia Appendix 1 The questionnaire.

## Abbreviations

EHR: electronic health record
