# Supplementary material for: Patients' Experiences of Web-Based Access to Electronic Health Records in Finland: Cross-sectional Survey
Source: J Med Internet Res. 2022 Jun 6;24(6):e37438. doi: 10.2196/37438 (PMC9210208; doi:10.2196/37438)
Supplement: Multimedia Appendix 1 [file jmir_v24i6e37438_app1.docx]

Multimedia Appendix A. The questionnaire.

1. What was the main reason for logging in to Oma Kanta this

time?

I wanted to:

Renew a prescription

View the information regarding prescriptions

View the results of physical examinations

View the medical notes

Give or withdraw consents data sharing

View log data related to data sharing

Give or edit my organ donation testament

Give or edit my living will

Use the patient portal on behalf of a minor

Use the patient portal on behalf of another adult

View the wellness data uploaded by using a wellness application

Something else, what?

2. Was your visit in Oma Kanta successful?

Yes, why?

No, why?

Do not know

3. Based on your experience, please evaluate the following

statements regarding Oma Kanta: (Scale from 1=strongly disagree to 7=

strongly agree, 8 = Do not know)

The capabilities of Oma Kanta meet my needs.

Using Oma Kanta is a frustrating experience

Oma Kanta is easy to use

4. How useful would you evaluate the following features of

Oma Kanta? (Scale: Have not used, Totally useless, Somewhat useless, Not

useless nor useful, Somewhat useful, Very useful)

Renew a prescription

Viewing the information regarding prescriptions

Viewing the results of physical examinations

Viewing the medical notes

Give or withdraw consents related to giving out data

Give or edit my organ donation testament

Give or edit my living will

Use the patient portal on behalf of a minor

Use the patient portal on behalf of another adult

Viewing the wellness data uploaded by using a wellness application

If the participant evaluated viewing the medical notes useful:

5. You evaluated viewing the notes written by a professional

about your health care visits useful. How would you describe the

benefits?

AND

6. Are there some challenges related to viewing the notes? How

would you describe them?

If the participant evaluated viewing the medical notes useless:

7. You evaluated viewing the notes written by a professional

about your health care visits fairly useless. What challenges are

related to viewing the notes?

OR

8. You evaluated viewing the notes written by a professional

about your health care visits completely useless. What challenges

are related to viewing the notes?

9. Have you discussed the notes about health care visits with a

doctor or another health care professional?

Yes

No

10. How could Oma Kanta be improved? You can also give ideas

for further development.

11. Have some of the following guided you to read the notes of

your health care visits written by a professional?

Physician or another health care professional

A written guide in health care

Family or friends

Kanta website

Newspapers, radio, TV, social media

Something else

12. How often have you used Oma Kanta lately?

Daily

Weekly

Monthly

Less often than monthly

This was the first time

13. Your age

Less than 18 years

18-35

36-50

51-65

66-75

76-85

86 years or older

14. Your gender

Female

Male

Other

Prefer not to say

15. Which device did you use to access Oma Kanta this time?

Computer

Smartphone

Tablet

Something else
